# Female mate preferences for male body size and shape promote sexual isolation in threespine sticklebacks

**DOI:** 10.1002/ece3.631

**Published:** 2013-06-05

**Authors:** Megan L Head, Genevieve M Kozak, Janette W Boughman

**Affiliations:** 1 Centre for Ecology and Conservation, School of Biosciences, University of ExeterCornwall Campus, Penryn, TR10 9EZ, UK; 2 Department of Animal Biology, University of IllinoisChampaign, Illinois, 61820; 3 Department of Zoology, Ecology, Evolutionary Biology and Behavior Program, BEACON, Michigan State UniversityEast Lansing, Michigan, 48824

**Keywords:** Magic traits, mate choice, reproductive isolation, sexual selection, speciation, Species recognition, threespine stickleback

## Abstract

Female mate preferences for ecologically relevant traits may enhance natural selection, leading to rapid divergence. They may also forge a link between mate choice within species and sexual isolation between species. Here, we examine female mate preference for two ecologically important traits: body size and body shape. We measured female preferences within and between species of benthic, limnetic, and anadromous threespine sticklebacks (*Gasterosteus aculeatus* species complex). We found that mate preferences differed between species and between contexts (i.e., within vs. between species). Within species, anadromous females preferred males that were deep bodied for their size, benthic females preferred larger males (as measured by centroid size), and limnetic females preferred males that were more limnetic shaped. In heterospecific mating trials between benthics and limnetics, limnetic females continued to prefer males that were more limnetic like in shape when presented with benthic males. Benthic females showed no preferences for size when presented with limnetic males. These results show that females use ecologically relevant traits to select mates in all three species and that female preference has diverged between species. These results suggest that sexual selection may act in concert with natural selection on stickleback size and shape. Further, our results suggest that female preferences may track adaptation to local environments and contribute to sexual isolation between benthic and limnetic sticklebacks.

## Introduction

Mate preferences for ecologically relevant traits have important consequences for local adaptation and speciation. Females often prefer mates that are in good phenotypic condition or that express traits which indicate high genetic quality (Iwasa and Pomiankowski 1991; Kirkpatrick and Ryan 1991; Pfennig 1998; Andersson and Simmons 2006). This is because these males can provide benefits to females that are either direct, such as increased fertility or parental care (Price et al. 1993; Kirkpatrick 1996; Moller and Jennions 2001) or indirect, such as genes that confer high fitness for offspring (Fisher 1930; Zahavi 1975). Many ecologically relevant traits may be reliable signals of a male's ability to acquire resources (Snowberg and Benkman 2009). Therefore, female preferences for traits that indicate male condition or genetic quality may target traits that are directly involved in adaptation to local environments (Lorch et al. 2003; Servedio 2004). This is because well-adapted males are likely to be of the best condition and quality. In this way, preferences for ecologically relevant traits may potentially drive local adaptation (sensu Kawecki and Ebert 2004) between populations.

Female preferences for ecologically relevant traits may also be important for the evolution of reproductive isolation and speciation (Servedio 2004; van Doorn et al. 2009). When populations or species adapt to new environments, natural selection will drive divergence in ecologically relevant traits generating either direct or indirect selection on female mate preferences if they are based on traits that are important in ecological adaptation. Thus, female preferences can track ecological divergence resulting in divergent preferences (Servedio 2004). When diverging populations subsequently come into contact, preferences for ecologically relevant traits will lead females to discriminate against males which have adapted to a different environment, generating sexual isolation (van Doorn et al. 2009). Therefore, preferences for ecologically relevant traits or well-adapted mates provide a way to link divergent natural selection with divergence in mating traits and mate preferences, which greatly facilitates ecological speciation even in the face of gene flow (Gavrilets 2005; Schluter and Conte 2009; Servedio et al. 2011).

Traits that are involved in both divergent selection due to adaptation to different environments and assortative mating between species have been termed ‘magic traits’ (Gavrilets 2004). Magic traits may facilitate speciation because the two functions of these traits are pleiotropically linked and cannot become disassociated via recombination (Servedio et al. 2011). Typically, magic traits are thought to arise through divergent natural selection acting on traits that are also used as mating cues, such as color or body size (e.g., Jiggins et al. 2001; Podos 2001). Putative magic traits have been identified in a number of species pairs (Servedio et al. 2011; Nosil 2012). However, in most cases, the basis of assortative mating and origin of female preferences for these traits is unknown. Sexual selection within species for well-adapted males could provide one mechanism to generate a preference for traits under natural selection (Servedio et al. 2011). The combined force of natural and sexual selection acting on preference for ecologically relevant traits may make these preferences a powerful driver of speciation (Ritchie 2007; Maan and Seehausen 2011).

**Figure 1:**
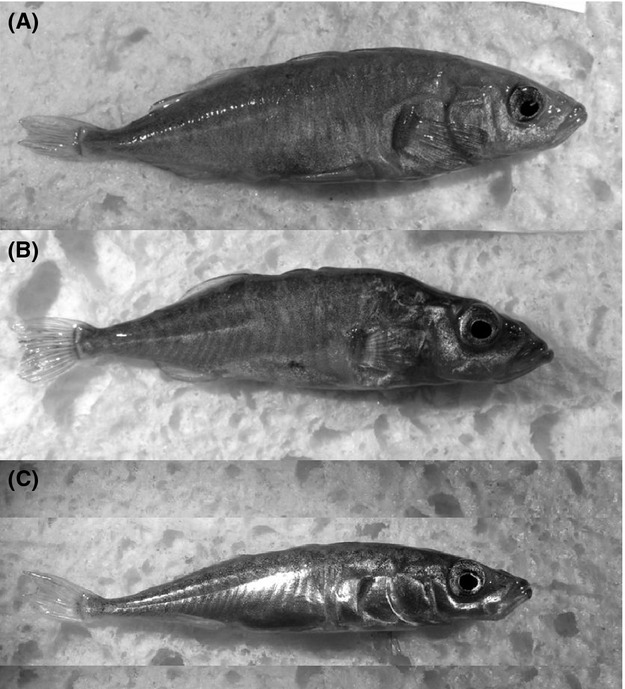
The three-spine stickleback species used in our study. (A) Anadromous male, (B) benthic male, (C) limnetic male.

Here, we investigate female mate preferences for two ecologically important traits, body shape and body size which have diverged in the threespine stickleback species complex (*Gasterosteus aculeatus* spp.) (Fig. 1). In the benthic–limnetic stickleback species complex, an extensive body of work has demonstrated that size and shape differences in sticklebacks result from adaptation to local foraging and predator environments (reviewed in McKinnon and Rundle 2002; Reid and Peichel 2010). Divergent natural selection has led to local adaptation of body shape and body size at two levels. First, benthic and limnetic sticklebacks have diverged from their anadromous ancestors by adapting to freshwater environments (McPhail 1994; Taylor and McPhail 2000; Walker and Bell 2000; Aguirre 2009). In general, anadromous fish are larger, have shorter heads and larger keels than freshwater fish; adaptations for long distance migration and predator avoidance in anadromous fish which are subsequently lost upon the colonization of freshwater environments (Taylor and McPhail 1986; McPhail 1994; Walker 1997; Walker and Bell 2000; Dalziel et al. 2012). Second, in freshwater lakes with extensive littoral and pelagic habitats, benthics and limnetics have diverged from each other in body size and shape during adaptation to different niches (benthic sticklebacks are specialized for feeding on macroinvertebrates in the littoral zone and limnetic sticklebacks are specialized for feeding on plankton in the pelagic zone), thus showing fine scale local adaptation. Evidence that differences in body size and shape between benthics and limnetics arise from adapting to different ecological niches is extensive. Benthics are large, deep-bodied, with a wide gape; this shape makes them highly maneuverable (Webb 1984; Walker 1997; Blake 2004) and enables them to forage efficiently on invertebrates located on the sediment or vegetation (Schluter 1993) and escape their grasping sit and wait predators (Walker 1997). In contrast, limnetics are small, slender, with a narrow upturned mouth; this shape gives them good prolonged swimming ability (Webb 1984; Walker 1997; Blake 2004) and enables them to forage efficiently on zooplankton in the open water and escape their pursuit predators (McPhail 1984, 1992; Malmquist 1992; Schluter and Mcphail 1992).

Body shape and body size make ideal candidates for female preferences based on ecologically relevant traits due to their role in adaptation to different foraging niches and to different suites of predators. What gives rise to female preferences for size remains poorly understood despite the fact that body size has been proposed as a putative magic trait in sticklebacks, under both natural and sexual selection (McKinnon et al. 2004; Boughman et al. 2005; Conte and Schluter 2013). Body size has been shown to be important in reproductive isolation between benthics and limnetics, with heterospecific mating being more common between similar sized individuals (Nagel and Schluter 1998; Boughman et al. 2005; Conte and Schluter 2013). However, females do not show preferences for body size within species (Head et al. 2009), suggesting that size-based assortative mating is not an outcome of sexual selection on size within species. One alternative is that size-based assortative mating may have evolved in response to selection on correlated characters such as body shape. Surprisingly, the role of shape for within or between species mate choice has not been considered in sticklebacks despite its clear role in adaptation and likely link with body size.

Our aims are threefold. First, to set the stage for further analyses, we confirm body shape and size differences between benthic and limnetic sticklebacks and their anadromous ancestor that have been found in previous studies (e.g., McPhail 1992; Taylor et al. 2005). Next, we use this information to investigate the relationship between body shape and size and determine whether this relationship is the same for all species. This analysis is important in the context of the current study, as body size and shape are tightly linked traits and one could evolve as a correlated response to selection on the other, or selection may act on combinations of these traits. Identifying the specific traits under selection aids our understanding of preference evolution and its contribution to sexual isolation (Servedio 2004; Servedio et al. 2011). Finally, we look at female preferences for body shape and size in both conspecific and heterospecific mating trials, to gain insight into the evolutionary processes that lead to reproductive isolation between the species. Thus, we ask if anadromous, benthic, and limnetic females have preferences for body size, body shape, or some combination of these traits. We test if these preferences are the same in all species or if they have diverged between species. We also investigate whether benthic and limnetic preferences lead to sexual isolation by comparing female preferences when choosing mates within and between species. In this way, we can determine if females have preferences for size and shape, traits clearly involved in adaptation, and whether these preferences may contribute to reproductive isolation.

## Methods

### Collection and maintenance of fish

Limnetic and benthic sticklebacks were collected from Paxton Lake (49^o^43′N, 124^o^31′W), Texada Island, British Columbia in April 2006 and 2007. During collection the assignment of fish to each species was based on a range of visual characteristics including shape, color, and size. All fish were easily assigned as either benthics or limnetics and no intermediate (hybrid) fish were caught. Anadromous sticklebacks were collected from the Nanaimo River (49^o^08′N, 123^o^54′W), Vancouver Island, British Columbia in May 2006. Once caught fish were shipped back to our lab in Madison, WI, where they were housed in single sex, 102 L tanks, in densities of up to 30 fish per tank. All fish were fed bloodworms (*Chironomus* sp.) and brine shrimp (*Artemia* sp.) daily and experienced a light:dark cycle of 14:10.

### Measuring female mate preferences

Within species no-choice mating trials were conducted in 2006 on all three species (benthic, limnetic, and anadromous) and between species no-choice mating trials were conducted between benthics and limnetics in 2007. The methods for these trials were very similar; however, where differences occur this is noted. These differences (in tank size and trial length) arise because within and between species trials were conducted in different years with different logistic constraints. Experimental studies looking at mate choice in threespine sticklebacks employ a wide range of methods and yet results tend to be consistent across studies (e.g., Künzler and Bakker 2001; Pike et al. 2007; Kozak et al. 2013). We use a standardized behavioral testing protocol and previous studies from our lab group have been performed across multiple years and never found a significant effect of year on female preference (Kozak et al. 2009, 2011, 2013). This suggests that the study of mate preferences in this species is robust to small differences in experimental setup and potential interannual variation.

Males that showed breeding coloration and behavior indicating readiness to breed were selected from single sex stock tanks for the experiment. Individual males were placed in trial tanks (intraspecific trials – 140 L; interspecific trials – 101 L) with nesting material and were enticed daily for 20 min with a gravid female in a jar to encourage nesting. Once males had completed a nest and were actively courting they were used in behavioral trials. Gravid females to be used in behavioral trials were identified from stock tanks daily by looking for females with distended abdomens. Reproductive state was verified by gently squeezing the abdomen of these females to look for ripe eggs in the oviduct. Gravid females were then stored in individual 5 L tanks with gravel and aeration to recover for a minimum of 1 h before being used in behavioral trials.

Female mating preferences for male body shape and size were assessed separately for con- and heterospecifics in no-choice mating trials. No choice trials are standard for studies looking at mating preferences carried out in sticklebacks (Hatfield and Schluter 1996; Nagel and Schluter 1998; Head et al. 2009). To begin a trial, a single gravid female was placed in a release container just below the surface of the water. Females were allowed 2 min to acclimate before being released into the male's aquarium. Behavioral observations began as soon as the male oriented toward the female and lasted 20 min (for intraspecific trials) or 10 min (for interspecific trials), or until the female entered the male's nest.

We recorded male and female behavior from approximately one meter in front of the aquarium using an event recorder (Observer – Noldus Technologies, Wageningen, the Netherlands). Male and female courtship behaviors recorded included zig-zagging, biting, leading, and following (described in Rowland 1994). From these behaviors we calculated female preference for a male as the proportion of male leads that resulted in the female following (Head et al. 2009; Kozak et al. 2009). Trials in which males did not perform any leads were excluded from analysis. We chose the proportion of follows per lead as our measure of female preference rather than measures occurring later in the courtship sequence (e.g., nest examination, spawning), because this measure controls for differing levels of courtship behavior which males may exhibit toward females (thus we do not confound our measure of female preference with male preference). Also, when females examine a male's nest they may gain further information about nest quality on which to base their mate choice decisions. By using a measure of female preference that occurs early in the courtship sequence we restrict the cues on which a female can base her mate choice decisions to those having to do with the male himself rather than an extended phenotype (the nest). Using the number of follows per lead also allows us to directly compare anadromous, benthic, and limnetic preferences while controlling for slight differences between species in the level of male courtship and amount of courtship that occurs before spawning as well as differences between trial lengths. Benthic males lead slightly less than limnetic and anadromous males and benthic and limnetic males lead slightly less to heterospecifics than to conspecifics, but these differences are not significant. In addition, anadromous fish spawn faster than benthics and limnetics (Head et al. 2009). We found that the number of female follows per lead is not related to the number of male leads (across all trials: *N* = 199, *r* = 0.114, *P* = 0.110), suggesting that females did not base their mate choice decisions on male courtship rate. Other stickleback studies have used earlier stages of courtship (Bakker and Milinski 1991; Conte and Schluter 2013) or later stages (Nagel and Schluter 1998; McKinnon et al. 2004). Previous work on benthic and limnetic sticklebacks suggests that sexual isolation is evident at any stage of courtship (Rundle and Schluter 1998; Kozak et al. 2009).

### Measuring male morphology

**Figure 2:**
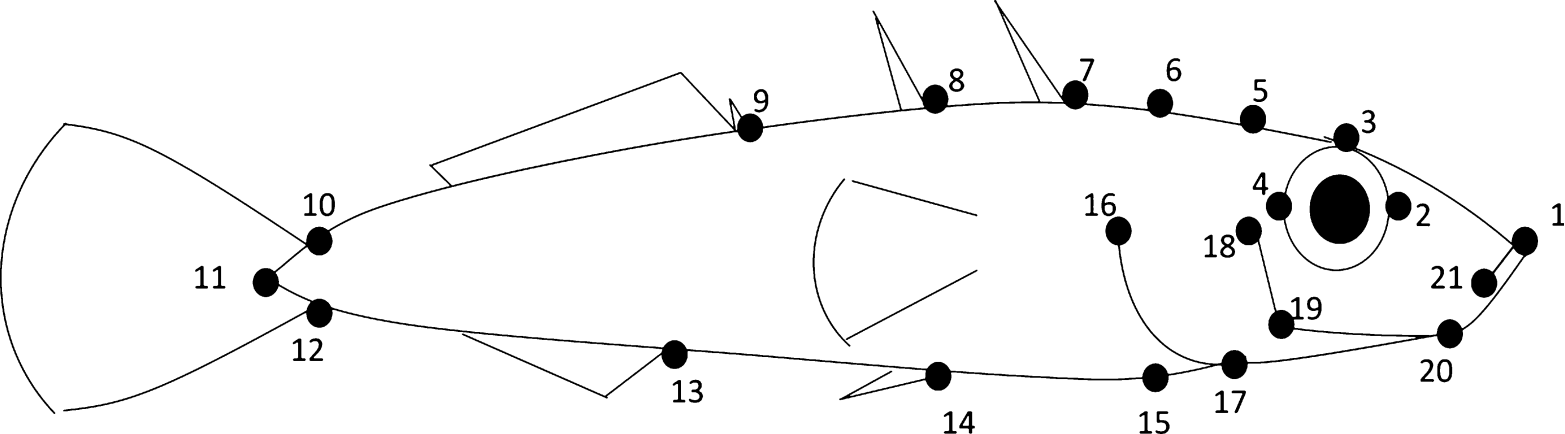
The 21 morphological landmarks recorded for morphological analysis: (1) anterior of the upper lip; (2) most anterior point of the right eye; (3) most dorsal point of right eye; (4) most posterior point of right eye; (5) midpoint of the line posterior to the top of the eye and the intersection with dorsal midline; (6) point of intersection between the dorsal midline and the line posterior to the top of the eye; (7) anterior junction of the first dorsal spine with the dorsal midline; (8) anterior junction of the second dorsal spine with the dorsal midline; (9) anterior insertion of the anal fin membrane with the dorsal midline; (10) insertion of the caudal fin on the dorsal midline; (11) caudal border of hypural plate at the lateral midline; (12) insertion of the caudal fin on the ventral midline; (13) anterior insertion of anal fin membrane with the ventral midline; (14) the point directly below landmark 8 on the ventral midline, a line between landmark 8 and 14 would be perpendicular to the lateral midline; (15) point directly below landmark 6 on the ventral midline, a line between landmarks 6 and 15 would be perpendicular to the lateral midline; (16) posteriodorsal extent of operculum; (17) posterioventral extent of preopercular; (18) dorsal point of angular; (19) posterior edge of angular; (20) anterior edge of angular; (21) posterior extent of maxilla.

Male shape and centroid size (i.e., the square root of the sum of squared distances of the landmarks from their centroid (Bookstein 1991) were measured from digital photographs taken of the right side of live fish. This removes the need to account for warping and bending that often occurs in studies of preserved fish. Images were imported into the program TPSDIG (Rohlf 1997) in which the location of 21 landmarks were recorded (Fig. 2). These landmarks were chosen for their accuracy of measurement and because they have been shown to capture shape variation between stickleback species previously (Walker and Bell 2000; Taylor et al. 2005). While previous work investigating reproductive isolation and mate preferences in sticklebacks use standard length as a proxy for body size (Nagel and Schluter 1998; McKinnon et al. 2004; Head et al. 2009; Conte and Schluter 2013), centroid size offers a more accurate shape independent measure of body size and is more commonly used in morphometric studies (Sanger et al. 2011; Wund et al. 2012).

### Data analysis

We used geometric morphometric methods for analyzing male shape. These methods have been reviewed extensively (Adams et al. 2004; Maderbacher et al. 2008) and are useful for the analysis of divergence in shape between populations in both sticklebacks (Albert et al. 2008; Aguirre 2009), other fish species (Parsons et al. 2003; Postl et al. 2008), and other taxa (e.g., damselflies – Outomuro et al. 2012; lizards – Sanger et al. 2011). We analyzed the shape of males using relative warp analysis (Rohlf 1999). We followed the methods of previous papers which have used this program (e.g., Taylor et al. 2005; Leinonen et al. 2006). Landmark coordinates for each fish were imported into the program TPSRELW (Rohlf 1997). This program uses Procrustes method to standardize each fish to a common size (with a centroid size of 1), as well as center and align the landmarks so that differences in size or positioning of the male do not contribute to shape differences between the males. The TPSRELW program then calculates a consensus configuration from the standardized coordinates. The program then compares each set of coordinates to the consensus configuration using thin plate spline analysis (Bookstein 1991). The method deforms each set of coordinates toward the consensus configuration, producing a unique set of energy values called ‘partial warps’. The principal components of these partial warps, called ‘relative warps’, summarize the major trends of shape variation in the set of specimens (Rohlf 1999). We conducted a single shape analysis for all males from both conspecific and heterospecific mate choice trials. This means that all males are scored along the same axes of shape and size variation.

We then performed canonical discriminant function analysis on the relative warp scores to assess overall shape differentiation among species and find the shape axes that best discriminated between the three species (Neves and Monteiro 2003; Taylor et al. 2005; Leinonen et al. 2006). We then used these discriminant function scores to look at the role of size in determining species shape differences. To do this we used ANCOVA with the first two discriminant functions specified as dependent variables, species specified as a fixed effect and centroid size specified as a covariate. This analysis allowed us to determine the effect of species, size, and the interaction between them on shape differences between the species. Nonsignificant terms were dropped from these models to produce the simplest best fitting models.

To determine whether male shape is important in female mate choice decisions we analyzed female preference for body shape, body size, and the interaction between them. This was done separately for conspecific and heterospecific mating trials, because of differences in the methods of data collection. In these analyses female response (the number of female follows per male lead) was included as the dependent variable, species was specified as a fixed factor and centroid size and the first and second discriminant functions were specified as covariates. We included all interaction terms in the full model and dropped nonsignificant terms sequentially to find the simplest, best fitting model for the data (Crawley 2007). We explored quadratic and correlational terms in our initial models but as these were not significant they were subsequently dropped from our analyses. Our response variable, the number of female follows per male lead, was standardized (to a mean of zero and a standard deviation of one) within species so that differences in the level of female responsiveness between species did not interfere with estimates of female preference. On average, anadromous females are more responsive than limnetics, who are more responsive than benthics (Head et al. 2009). All predictor variables were normally distributed (determined from visualizing Q-Q plots) and were standardized to units of standard deviation from the mean phenotype within species to allow direct comparison of female preferences among traits and species. Standardization of both the response and predictor variables was done separately for the two different types of mating trials (i.e., conspecific and heterospecific). Two anadromous outliers were excluded from our analysis because females were over-responsive (following males far more often than they were led). The removal of these females did not qualitatively change our results. For conspecific trials sample sizes were anadromous: *N* = 53, benthic: *N* = 43, limnetic: *N* = 43. For heterospecific trials sample sizes were benthic males: *N* = 30, limnetic males: *N* = 32. All statistical analyses were performed using SPSS (version 16).

## Results

### Body shape and size

**Figure 3:**
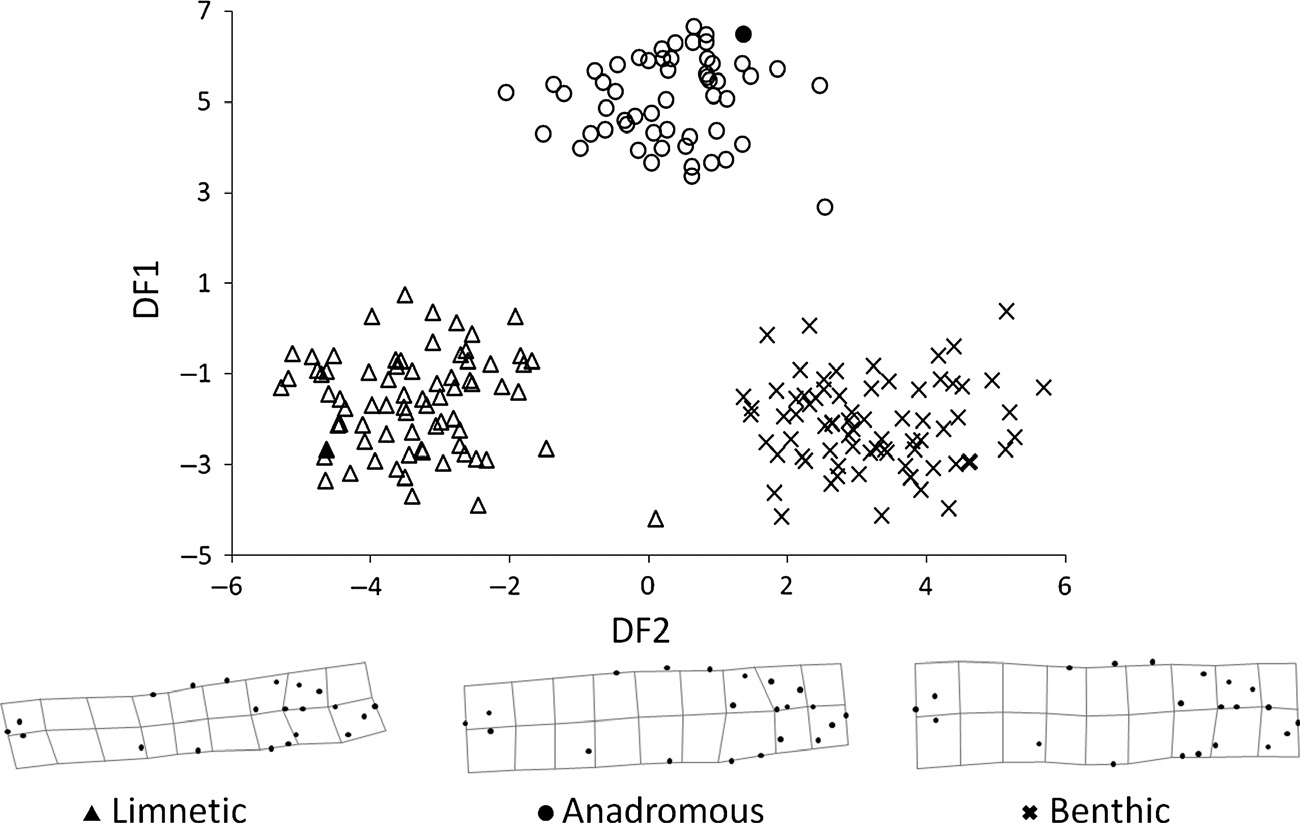
Three species plotted along the two major axes of shape differentiation between the species. Circles: anadromous males; crosses: benthic males; triangles: limnetic males. Both discriminant functions differ significantly between species (DF1: χ^2^ = 832.162, *P* < 0.001; DF2: χ^2^ = 400.886, *P* < 0.001). Thinplate spline plots indicate the deviation of each species from the consensus figure, males shown in thinplate splines are represented by bold symbols.

The canonical discriminant function analysis identified two major axes of shape variation. The first axis of shape variation (DF1) explains 54.6% of body shape differentiation among species, and shows that anadromous males have deeper anterior body regions and shorter heads than either of their lake counterparts. The second discriminant function (DF2) explains 45.4% of shape differentiation among species and shows that benthics and limnetics have diverged in body shape from their anadromous ancestor. Benthic males are deeper along their entire body (caudal peduncle, midsection, and head) and have more forward pointing jaws while limnetic males have shallower bodies and more upturned jaws (Fig. 3). Relative warps and how they contribute to each discriminant function are given in Table S1, Table S2, Fig. S1.

Males also differed significantly in body size (mean centroid size ± S.E. – anadromous: 2501.99 ± 18.39; benthic: 2429.18 ± 29.55; limnetic: 2269.44 ± 33.54, *F*_2,201_ = 15.900, *P* < 0.001). Limnetic males were smaller than both anadromous males and benthic males (Tukey's test: *P* < 0.001, *P* < 0.001, respectively), whereas anadromous males and benthic males did not differ (Tukey's test: *P* = 0.217). Species differences in body size were similar whether we analyzed centroid size or standard length (mean standard length ± S.E.: anadromous – 56.53 ± 0.36; benthic – 56.49 ± 0.67; limnetic – 48.97 ± 0.47, *F*_2,201_ = 67.082, *P* < 0.000). Centroid size and standard length are correlated for anadromous (*r* = 0.659, *P* < 0.001) and limnetic (*r* = 0.371, *P* = 0.001) sticklebacks but not benthics (*r* = 0.161, *P* = 0.171).

**Figure 4:**
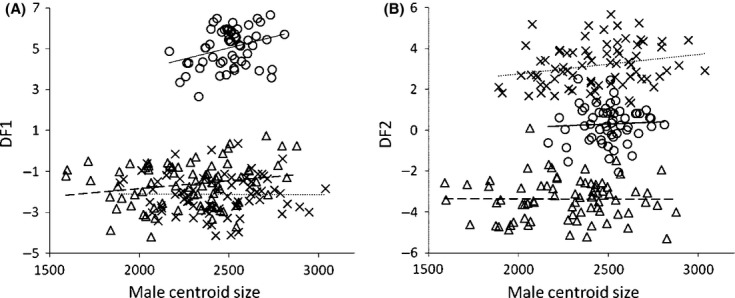
The relationship between species, centroid size and (A) the first discriminant function describing body shape differences between the species (DF1) and (B) the second discriminant function describing body shape differences between the species (DF2). Circles (solid line): anadromous males; crosses (dotted line): benthic males; and triangles (dashed line): limnetic males. For DF1 the interaction term was nonsignificant (*F*_2,198_ = 2.348, *P* = 0.098) and was thus excluded from the final model. Both species and centroid size explained a significant amount of the variation in male body shape (*F*_2,200_ = 904.189, *P* < 0.001; *F*_1,200_ = 3.975, *P* = 0.048; respectively). For DF2 there was no effect of the interaction between species and centroid size (*F*_2,198_ = 1.239, *P* = 0.292), nor of centroid size alone (*F*_1,200_ = 1.945, *P* = 0.165) and so these terms were excluded from the final model. Species explained a significant amount of the variation in male body shape (*F*_2,201_ = 803.465, *P* < 0.001.

Our analysis of how species and body size influence shape revealed an overall positive relationship between DF1 and centroid size (Fig. 4A). This shows that larger fish tend to have deeper anterior body regions and shorter heads than smaller fish, however, this pattern appears to be driven mostly by a relationship within the anadromous fish (β = 0.313, *r*^2^ = 0.098, *P* = 0.020) and to a lesser extent by limnetics (β = 0.210, *r*^2^ = 0.004, *P* = 0.071), while benthic sticklebacks show no relationship (β = −0.010, *r*^2^ = 0.000, *P* = 0.930). In addition, a significant effect of species on DF1 indicates that species differences in body shape are not simply an extrapolation of a common allometric curve (i.e., individuals from the different species are not a different shape simply because they differ in size) (Fig. 4A). For DF2, there was a strong effect of species but no effect of body size on shape (Fig. 4B). This indicates that body size does not account for differences in overall body depth or jaw angle either within or among the species.

### Female preference when assessing conspecific males

**Figure 5:**
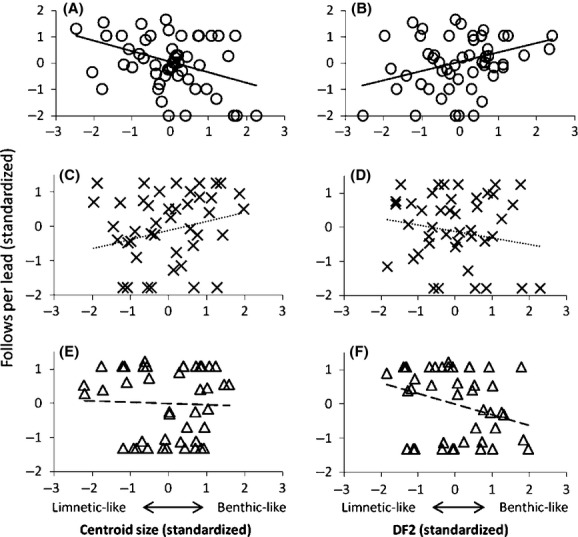
Female within-species mate preferences for male body shape and size. (A and B) Show anadromous female preferences for centroid size and DF2, respectively. Anadromous females prefer small anadromous males (β ± SE = −0.400 ± 0.139) that are benthic like in shape (β ± SE = 0.351 ± 0.127). (C and D) show benthic female preferences for centroid size and DF2, respectively. Benthic females prefer large benthic males (β ± SE = 0.265 ± 0.171), but show little preference for shape (β ± SE = −0.190 ± 0.183). (E and F) Show limnetic female preferences for centroid size and DF2, respectively. Limnetic females show no preference for size of limnetic males (β ± SE = −0.034 ± 0.160), but prefer males that are more limnetic like in shape (β ± SE = −0.309 ± 0.171). Because ANCOVA revealed species differences in female preferences for body size and shape, regression slopes for within-species preferences were obtained from within-species multiple regression.

**Figure 6:**
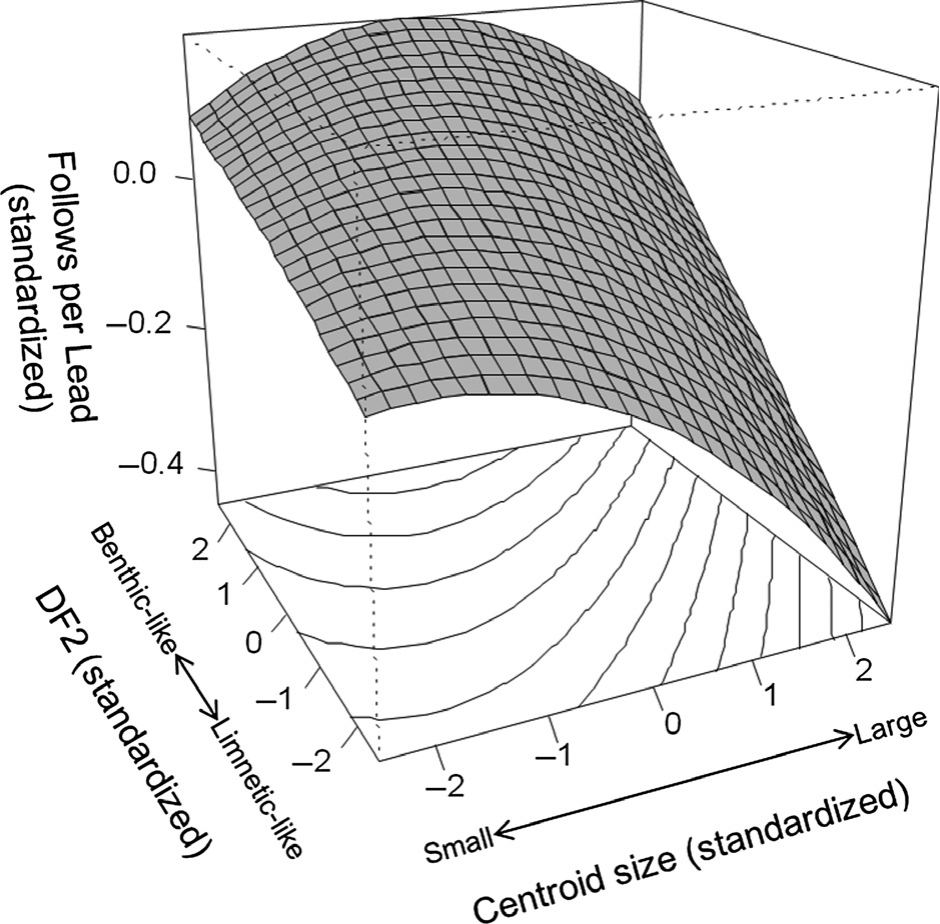
Response surface showing anadromous female mate preferences for centroid size and DF2.

Females of the three species differed in their preferences for male size and shape when assessing males of their own species. The final model of the effects of species, size, and shape on female preferences revealed a significant three-way interaction between species, size, and DF2 (*F*_2,122_ = 3.432, *P* = 0.035), as well as two-way interactions between species and size (*F*_2,122_ = 4.585, *P* = 0.012) and species and DF2 (*F*_2,122_ = 5.490, *P* = 0.005) (Fig. 5). This interaction is at least partly due to mate preference differences between benthics and limnetics, because the three-way interaction between species, size, and DF2 remained significant when anadromous fish were excluded from our analysis (*F*_1,74_ = 5.414, *P* = 0.023). On closer inspection, our results show that anadromous females base mate choice decisions on both body size and shape, appearing to dislike large and slender anadromous males (Fig. 6). In contrast, for the traits we measured benthic females base mate choice decisions predominately on size, preferring larger males of their own species, while limnetic females base mate choice decisions predominately on shape, preferring more limnetic-shaped males of their own species (Fig. 5).

### Female preference when assessing heterospecific males

**Figure 7:**
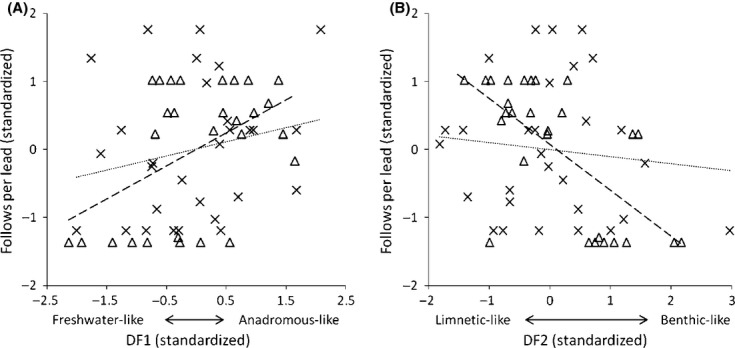
Between species mate preferences of benthic and limnetic females based on shape. Triangles (dashed line): Limnetic females assessing benthic males, Crosses (dotted line): Benthic females assessing limnetic males. (A) shows female preference based on DF1 revealing that limnetic females prefer males that are more anadromous like (β ± SE = 0.336 ± 0.141) while benthic females show no preference (β ± SE = 0.198 ± 0.200). (B) shows female preference based on DF2 revealing that limnetic females prefer benthic males that are more limnetic like in shape (β ± SE = −0.580 ± 0.142), while again benthic females show no preference along this axis of shape variation (β ± SE = −0.043 ± 0.194).

Benthic and limnetic females also differed in their preferences when assessing heterospecific males. In the final model of the effects of species, size, and shape on between species preference there was a significant interaction between species and DF2 (*F*_1,52_ = 6.527, *P* = 0.014) as well as main effects of both DF1 (*F*_1,52_ = 4.721, *P* = 0.034) and DF2 (*F*_1,52_ = 6.717, *P* = 0.012). Contrary to expectation, size was not important in determining female preferences between species (all terms including size were nonsignificant and dropped from the final model: all *F* < 0.904, *P* > 0.346). Our final model reveals that limnetic females prefer benthic males that are more limnetic-like along the shape axis DF2 (Fig. 7B) and males that are more anadromous like along DF1 (Fig. 7A). In contrast, benthic females show no preference for shape when assessing heterospecific males.

## Discussion

How females use ecologically relevant traits when choosing mates helps us understand how natural and sexual selection may interact to cause speciation (Ritchie 2007; Maan and Seehausen 2011). Body size and shape in sticklebacks are known adaptations to foraging and escaping predators in different environments (reviewed in introduction). Here, we show that stickleback females have preferences for male body size, shape, or a combination of the two, but that these preferences differ depending on the females’ own species identity. When assessing conspecific males, anadromous females base their mate choice on a combination of size and shape. Conspecific preferences have diverged from this ancestral preference in both benthic and limnetic sticklebacks. Benthic females choose mates based predominantly on size while limnetics choose mates based predominantly on shape. Therefore, our results from conspecific trials suggest that stickleback females do have mate preferences for ecologically relevant traits, but these differ between populations. Our results from heterospecific trials suggest that limnetic preferences for shape are used to discriminate against benthic males and thus shape preferences contribute to sexual isolation. This preference for shape is an incomplete isolating barrier, but likely contributes to strong sexual isolation between species when combined with preferences for other traits such as nuptial color and odor.

### Male body size and shape divergence between species

Our results show that there has been significant shape divergence between anadromous, benthic, and limnetic sticklebacks, confirming results of previous studies (e.g., Walker and Bell 2000; Aguirre 2009). A novel aspect of our shape analysis is that we show that male shape differences between species are not simply due to species differences in body size, and that allometric relationships between size and shape have also diverged. The differences in both the slope and intercept of allometry that we find between species suggests that divergence in body shape is unlikely to be caused by a correlated response to selection on body size (McGuigan et al. 2010). This result is important because distinguishing the patterns of variation and covariation in size and shape will help to identify the nature of selection on these traits, and how their evolutionary change affects reproductive isolation.

Species-specific relationships between size and shape also suggest there may be natural selection for certain shape/size combinations in fish, possibly due to locomotor performance. Positive allometry between DF1 and body size appears to be driven mostly by anadromous males (Fig. 4A), with larger males having relatively smaller heads and deeper caudal peduncles. This same relationship occurs in Alaskan anadromous sticklebacks (McGuigan et al. 2010; Wund et al. 2012). The fact that this allometric slope decreases in lake fish (particularly for sedentary benthics) may indicate that allometry is beneficial for long distance swimming performance (Webb 1984; Blake 2004). Such ecologically dependent relationships between shape and size may have important consequences for the evolution of reproductive isolation. Reduced hybrid performance in parental foraging niches is a key mechanism of postmating reproductive isolation in ecological speciation, and is known to be important in stickleback speciation (Schluter 1993; Hatfield and Schluter 1999; Rundle 2002). This may occur not only because hybrids have intermediate phenotypes (Schluter 1993) but also due to an uncoupling of adaptive trait combinations of body size with body shape or trophic morphology. Mismatched trait combinations reducing hybrid performance are likely to be important in a broad range of taxa including lizards (Lancaster et al. 2010) and even plants (Melendez-Ackerman and Campbell 1998).

### Anadromous female preferences for body size and shape

Anadromous stickleback females had preferences for both size and shape: females disliked anadromous males that were large and limnetic shaped. We hypothesize that this avoidance of large slender males may reflect a preference for males that are in good condition (because males that are “skinny” for their size are likely to be malnourished). Alternatively, slender males may be avoided because they are at a fitness disadvantage: because large fusiform bodies increase prolonged swimming performance required for migration (Walker 1997) and deep bodies decrease predation by gape limited predators (Reimchen 1994). Anadromous female preference may also reflect a combination of the two, because condition is likely to reflect local adaptation (Weissing et al. 2011). Future work examining the benefits that anadromous females who mate with smaller, deep-bodied males gain would help to solve this question.

Anadromous female mate preferences for body size and body shape may be key to the rapid morphological evolution seen when these fish colonize freshwater habitats. Anadromous populations of sticklebacks have colonized freshwater streams and lakes repeatedly throughout the Northern Hemisphere, resulting in freshwater populations with great variation in size and shape (e.g., Walker 1997; Leinonen et al. 2006; Spoljaric and Reimchen 2008). Many of the same phenotypes are seen repeatedly in similar habitats, implicating selection in their evolution. Further, several studies have shown that divergence in body size and shape can occur rapidly (within 8–11 generations – Aguirre and Bell 2012). Ancestral preferences for size and shape can complement divergent natural selection for these same traits, hastening the pace of evolutionary change because of dual effects of natural and sexual selection (Ritchie 2007) and coupling adaptation to assortative mating (Servedio 2004). These ancestral preferences also allow mate preferences to track natural selection in novel environments. Thus, mate preferences may enhance natural selection and lead to rapid adaptation to new environments (Fricke and Arnqvist 2007). This may be particularly important for increasing the likelihood of speciation when populations are geographically isolated for short periods of time before coming into secondary contact. Evidence from other species radiations also shows ecological divergence and mate preferences for ecologically relevant traits (e.g., beak size in the medium ground finch – Huber et al. 2007; color pattern in *Heliconius* butterflies – Merrill et al. 2011). This may indicate that mate preferences for ecologically relevant traits may provide a general mechanism for rapid divergence.

### Benthic and limnetic female preferences and sexual isolation

Benthic and limnetic mate preferences for body size and body shape have diverged from those of their anadromous ancestor and from each other. Essentially, each of these species continues to base mate choice decisions on these ecologically relevant traits, however, the relative importance of body size and body shape to mate choice have changed (benthic females focus on size, while limnetic females focus on shape). This suggests each species has diverged along a different axis of ancestral mate preference variation. There are several possible reasons for this divergence in preference between benthics and limnetics. One hypothesis is that mate preferences are for well-adapted mates and tracking traits that are most important in local adaptation in each species (Servedio 2004). Large, deep bodies play a key role in avoiding predation and navigating in benthic environments, so potentially benthic females benefit from preferring large males. Likewise, limnetic shape is an adaptation to foraging on plankton in the open water and limnetic females may benefit from preferring males with the most extreme and presumably well-adapted shape. Another reason for this difference between species may reflect differences in the reliability of these traits for signaling species identity. Benthics and limnetics live and mate in the same lakes, as such avoiding heterospecific mates is a constant problem. As benthics are larger and deeper bodied than limnetics, a preference for large males may enable avoidance of small limnetic males. For limnetics, on the other hand, female preferences based on size may lead to conflict between choice for good quality conspecific males and avoidance of heterospecifics (Pfennig 1998) if there is a relationship between quality and size and small limnetics are low quality. As such selection may target a female preference for shape which may reduce such conflict. These ideas deserve to be tested in future work, along with possible mechanisms that lead to female preferences for size and shape (e.g., phenotype matching [Conte and Schluter 2013] or learning [Kozak et al. 2011]).

Mate preferences for ecologically relevant traits may have important consequences for the evolution of reproductive isolation. This is because within-species preferences for such traits can be easily extended to between species mate choice and lead to assortative mating (Servedio 2004; van Doorn et al. 2009; Weissing et al. 2011). Limnetic female preference for limnetic-shaped males leads these females to reject benthics, particularly those that are very benthic like in shape. A preference for limnetic-shaped males therefore contributes to sexual isolation and provides the building blocks for the evolution of magic traits. However, within-species female preferences do not necessarily lead to the evolution of a magic trait. Unlike limnetic mate preferences for shape, benthic preferences for large body size did not extend to between species trials (despite overlap in the body size of limnetic and benthic males). This result contrasts to that found in previous studies, which suggested that body size is important for reproductive isolation between these stickleback species (Nagel and Schluter 1998; Boughman et al. 2005; Conte and Schluter 2013). Based on those studies, many have hypothesized that body size operates as a magic trait in benthic and limnetic sticklebacks. However, these studies used standard length (rather than centroid size) and did not control for differences in shape, as such, assortative mating based on size found previously may be confounded with differences in shape between species. Furthermore, body size may contribute to assortative mating through other mechanisms such as male mate choice (Kozak et al. 2009)) or mate choice for nest characteristics that correlate with body size (Wong et al. 2012). Investigating how traits that are correlated with body size affect assortative mating may aid in determining the role that body size plays in sexual isolation of these species as well as other sticklebacks species where manipulative experiments have shown that body size contributes to sexual isolation (e.g., McKinnon et al. 2004; Furin et al. 2012).

In sticklebacks, female preferences for multiple traits appear to be important for sexual isolation. Our result that limnetic female preferences for body shape contribute to sexual isolation adds to previous research which has found that color (Boughman 2001) and odor (Rafferty and Boughman 2006) are important for sexual isolation between benthic and limnetic sticklebacks. Interestingly, like for color and odor (Boughman 2001; Rafferty and Boughman 2006), the way in which shape contributes to sexual isolation is asymmetrical between species. Limnetic females are isolated from benthic males due to a combination of shape and nuptial color, while benthic females are isolated from limnetic males via preferences for odor. Work from other systems suggests that reproductive isolating barriers are typically asymmetrical, including both sexual isolation (Yukilevich 2012) and hybrid inviability (Bolnick et al. 2008; Schrader and Travis 2008). Thus, asymmetrical barriers may be the norm rather than the exception and reproductive isolation may typically accrues due to the combined action of many barriers.

## Conclusions

Here, we present several important findings that contribute to our understanding of how sexual selection can aid the evolution of reproductive isolation. We provide evidence that when assessing conspecific mates, females use ecologically relevant traits such as size and shape. Within-species mate choice for these traits under natural selection may contribute to the evolution of reproductive isolation in two important ways. First, when sexual selection and natural selection act in the same direction on the same traits, rapid divergence of these traits is expected. Second, these traits may act as magic traits in speciation, allowing for assortative mating to evolve between species. We suggest that body shape is such a magic trait in this stickleback system. Further studies of body size and shape preferences in other stickleback populations may be useful for determining the generality of our results.

## Supporting Information

**Table S1.** Multivariate and descriptive statistics from MANOVA for all relative warp scores of shape differences between species.

**Table S2.** Standardised canonical discriminant function coefficients. Loadings of each relative warp on the two major axes of shapes differentiation among species. Relative warps given in bold differed significantly between populations (see Table S1.) and the shape variation along these axes is shown below (Fig. S1).

**Figure S1.** Thin-plate spline plots indicating shape deviation from the consensus shape for each of the relative warps that showed differences between species. (A) Relative warp 1, high values indicate narrower bodies and elongated heads, (B) relative warp 2, high values indicate shorter heads, (C) relative warp 3, high values indicate deeper caudal peduncles and deeper bodies and deeper heads, (D) relative warp 4, high values indicate posterior movement of landmark 6, (E) relative warp 6, high values indicate anterior movement of landmark 7 and larger heads, (F) relative warp 8, high values show anterior movement of landmark 13, (G) relative warp 22, note the movement of landmark 21 relative to landmark 1, showing a difference in the angle of the mouth.
