# Supplementary material for: Female mate preferences for male body size and shape promote sexual isolation in threespine sticklebacks
Source: Ecol Evol. 2013 Jun 5;3(7):2183–96. doi: 10.1002/ece3.631 (PMC3728956; doi:10.1002/ece3.631)
Supplement: Supplementary file 1 [file ece30003-2183-SD1.docx]

**Supplementary material**

Table S1. Multivariate and descriptive statistics from MANOVA for all relative warp scores of shape differences between species.

| Relative warp | % of variation explained | Mean (x10^3^)  (S.E) | | | F | p |
| --- | --- | --- | --- | --- | --- | --- |
|  |  | Anadromous | Benthic | Limnetic |  |  |
| Multivariate |  |  |  |  | 37.958^a^ | 0.000 |
| 1 | 22.45 | -168.91  (2.0201) | -5.708  (1.657) | 18.019  (2.558) | 67.416 | 0.000 |
| 2 | 17.23 | 21.636  (1.676) | -4.895  (1.813) | -11.037  1.819 | 82.796 | 0.000 |
| 3 | 13.85 | -8.704  1.366 | 16.996  1.609 | -10.386  1.444 | 106.902 | 0.000 |
| 4 | 7.74 | 4.876  (1.366) | -6.071  (1.698) | 2.414  1.398 | 14.053 | 0.000 |
| 5 | 6.47 | 0.682  (1.538) | -0.540  (1.602) | 0.032  (1.309) | 0.156 | 0.856 |
| 6 | 5.64 | 3.338  (1.216) | -0.004  (1.559) | -2.444  (1.197) | 4.182 | 0.017 |
| 7 | 4.85 | -0.395  (1.168) | 0.387  (1.249) | -0.092  (1.365) | 0.089 | 0.914 |
| 8 | 3.38 | 0.392  (1.262) | 1.630  (1.012) | -1.896  (0.966) | 3.086 | 0.048 |
| 9 | 2.86 | 0.747  (0.984) | 0.403  (1.078) | -0.945  (0.871) | 0.824 | 0.440 |
| 10 | 2.09 | -1.650  (1.051) | 1.233  (0.784) | -0.007  (0.732) | 2.752 | 0.066 |
| 11 | 1.94 | 0.020  (0.823) | -1.046  (0.872) | 1.018  (0.714) | 1.780 | 0.171 |
| 12 | 1.59 | 0.651  (0.654) | 0.103  (0.804) | 0.376  (0.697) | 0.466 | 0.628 |
| 13 | 1.20 | 1.163  (0.657) | -0.757  (0.656) | -0.107  (0.587) | 2.140 | 0.120 |
| 14 | 1.07 | 0.755  (0.537) | -0.908  (0.614) | 0.342  (0.604) | 2.062 | 0.130 |
| 15 | 0.91 | -0.491  (0.478) | 0.077  (0.575) | 0.284  (0.569) | 0.465 | 0.629 |
| 16 | 0.81 | 0.459  (0.501) | -0.097  (0.605) | -0.241  (0.437) | 0.441 | 0.644 |
| 17 | 0.75 | -0.040  (0.414) | 0.282  (0.549) | -0.248  (0.489) | 0.303 | 0.739 |
| 18 | 0.62 | 0.146  (0.414) | -0.091  (0.486) | -0.018  (0.453) | 0.062 | 0.940 |
| 19 | 0.53 | 0.219  (0.442) | -0.053  (0.410) | -0.108  (0.421) | 0.150 | 0.861 |
| 20 | 0.47 | 0.028  (0.440) | -0.038  (0.387) | 0.017  (0.381) | 0.008 | 0.992 |
| 21 | 0.42 | 0.118  (0.368) | -0.067  (0.335) | 0.502  0.338 | 0.058 | 0.944 |
| 22 | 0.38 | 0.314  (0.410) | -0.743  (0.335) | 0.502  (0.336) | 3.773 | 0.025 |
| 23 | 0.36 | 0.061  (0.334) | 0.354  (0.387) | -0.394  (0.309) | 1.267 | 0.284 |
| 24 | 0.32 | 0.302  (0.315) | -0.176  (0.317) | 0.048  (0.339) | 0.508 | 0.602 |
| 25 | 0.27 | -0.307  (0.301) | 0.208  (0.289) | 0.020  (0.316) | 0.660 | 0.518 |
| 26 | 0.27 | 0.114  (0.291) | -0.090  (0.303) | 0.005  (0.301) | 0.105 | 0.900 |
| 27 | 0.22 | 0.196  (0.272) | -0.309  (0.265) | 0.161  (0.282) | 1.074 | 0.344 |
| 28 | 0.21 | -0.339  (0.278) | 0.482  (0.255) | -0.227  (0.267) | 2.811 | 0.062 |
| 29 | 0.19 | -0.010  (0.250) | 0.048  (0.270) | -0.040  (0.233) | 0.034 | 0.967 |
| 30 | 0.15 | -0.019  0.240 | -0.052  (0.238) | -0.037  (0.209) | 0.046 | 0.955 |
| 31 | 0.15 | 0.025  (0.255) | -0.190  (0.202) | 0.169  (0.220) | 0.711 | 0.493 |
| 32 | 0.13 | 0.096  (0.219) | 0.085  (0.214) | -0.155  (0.194) | 0.474 | 0.623 |
| 33 | 0.12 | 0.070  (0.222) | -0.110  (0.200) | 0.058  (0.182) | 0.259 | 0.772 |
| 34 | 0.10 | -0.021  (0.191) | 0.010  (0.171) | 0.006  (0.186) | 0.007 | 0.993 |
| 35 | 0.09 | -0.107  (0.198) | 0.078  (0.155) | 0.001  (0.173) | 0.262 | 0.770 |
| 36 | 0.07 | 0.052  (0.170) | -0.043  (0.156) | 0.004  (0.147) | 0.086 | 0.917 |
| 37 | 0.06 | 0.042  (0.151) | -0.027  (0.126) | -0.004  (0.155) | 0.054 | 0.948 |
| 38 | 0.06 | -0.026  (0.151) | 0.035  (0.128) | -0.015  (0.139) | 0.055 | 0.947 |

^a^ Multivariate statistic based on Pillai’s Trace

Table S2. Standardised canonical discriminant function coefficients. Loadings of each relative warp on the two major axes of shapes differentiation among species. Relative warps given in bold differed significantly between populations (see Table S1.) and the shape variation along these axes is shown below (Figs. S1).

| Relative Warp | DF1 | DF2 |
| --- | --- | --- |
| **1** | -1.112 | -1.168 |
| **2** | 1.645 | 0.414 |
| **3** | -0.809 | 1.401 |
| **4** | 0.768 | -0.762 |
| 5 | 0.120 | -0.056 |
| **6** | 0.574 | 0.322 |
| 7 | -0.082 | 0.057 |
| **8** | 0.052 | 0.539 |
| 9 | 0.173 | 0.236 |
| 10 | -0.504 | 0.208 |
| 11 | 0.036 | -0.415 |
| 12 | -0.218 | -0.075 |
| 13 | 0.466 | -0.137 |
| 14 | 0.337 | -0.318 |
| 15 | -0.217 | -0.075 |
| 16 | 0.217 | 0.059 |
| 17 | -0.033 | 0.172 |
| 18 | 0.082 | -0.021 |
| 19 | 0.128 | 0.029 |
| 20 | 0.019 | -0.022 |
| 21 | 0.080 | -0.015 |
| **22** | 0.256 | -0.544 |
| 23 | 0.018 | 0.352 |
| 24 | 0.236 | -0.050 |
| 25 | -0.260 | 0.085 |
| 26 | 0.099 | -0.046 |
| 27 | 0.199 | -0.268 |
| 28 | -0.344 | 0.407 |
| 29 | -0.015 | 0.057 |
| 30 | -0.026 | 0.063 |
| 31 | 0.048 | -0.262 |
| 32 | 0.102 | 0.196 |
| 33 | 0.098 | -0.133 |
| 34 | -0.029 | 0.002 |
| 35 | -0.160 | 0.063 |
| 36 | 0.088 | -0.045 |
| 37 | 0.076 | -0.021 |
| 38 | -0.052 | 0.057 |

Figure S1. Thin-plate spline plots indicating shape deviation from the consensus shape for each of the relative warps that showed differences between species. A) relative warp 1, high values indicate narrower bodies and elongated heads, B) relative warp 2, high values indicate shorter heads, C) relative warp 3, high values indicate deeper caudal peduncles and deeper bodies and deeper heads, D) relative warp 4, high values indicate posterior movement of landmark 6, E) relative warp 6, high values indicate anterior movement of landmark 7 and larger heads, F) relative warp 8, high values show anterior movement of landmark 13, G)relative warp 22, note the movement of landmark 21 relative to landmark 1, showing a difference in the angle of the mouth.
